# Dual Regulation of Phosphatidylserine Decarboxylase Expression by Envelope Stress Responses

**DOI:** 10.3389/fmolb.2021.665977

**Published:** 2021-05-07

**Authors:** Yasmine Hassoun, Julia Bartoli, Astrid Wahl, Julie Pamela Viala, Emmanuelle Bouveret

**Affiliations:** ^1^LISM, Institut de Microbiologie de la Méditerranée, UMR 7255, CNRS and Aix-Marseille Université, Marseille, France; ^2^SAMe Unit, UMR 2001, Microbiology Department, Pasteur Institute, Paris, France

**Keywords:** *psd*, *mscM*, SigmaE, CpxRA, envelope stress response, *E. coli*, phospholipid synthesis

## Abstract

Bacteria adapt to versatile environments by modulating gene expression through a set of stress response regulators, alternative Sigma factors, or two-component systems. Among the central processes that must be finely tuned is membrane homeostasis, including synthesis of phospholipids (PL). However, few genetic regulations of this process have been reported. We have previously shown that the gene coding the first step of PL synthesis is regulated by σ^E^ and ppGpp, and that the BasRS (PmrAB) two component system controls the expression of the DgkA PL recycling enzyme. The gene coding for phosphatidylserine decarboxylase, the last step in phosphatidylethanolamine synthesis is another gene in the PL synthesis pathway susceptible of stress response regulation. Indeed, *psd* appears in transcriptome studies of the σ^E^ envelope stress Sigma factor and of the CpxAR two component system. Interestingly, this gene is presumably in operon with *mscM* coding for a miniconductance mechanosensitive channel. In this study, we dissected the promoter region of the *psd-mscM* operon and studied its regulation by σ^E^ and CpxR. By artificial activation of σ^E^ and CpxRA stress response pathways, using GFP transcriptional fusion and western-blot analysis of Psd and MscM enzyme production, we showed that the operon is under the control of two distinct promoters. One is activated by σ^E^, the second is activated by CpxRA and also responsible for basal expression of the operon. The fact that the phosphatidylethanolamine synthesis pathway is controlled by envelope stress responses at both its first and last steps might be important for adaptation of the membrane to envelope perturbations.

## Introduction

Phospholipids (PL) are major structural and functional components of biomembranes and play a dynamic role in many regulatory processes. The biochemistry of the PL biosynthesis pathway is well described in bacteria (Parsons and Rock, 2013). It begins by two successive acylations of the positions 1 and 2 of a glycerol 3-phosphate backbone to give phosphatidic acid. Then, the third position of the future polar head group is activated with a CDP nucleotide. Finally, two separate pathways give rise in *Escherichia coli* to the zwitterionic phosphatidylethanolamine (PE), or to the anionic phospholipids phosphatidylglycerol (PG), and cardiolipin (CL). Bacterial species display specific membrane compositions, and the less frequent phosphatidylcholine, phosphatidylinositol, and a variety of other membrane lipids can be found in addition to the common PE, PG, and CL lipids (Sohlenkamp and Geiger, 2016).

Though the composition of the membrane is highly stable in *E. coli*, it can adapt to the growth conditions, through modification of fatty acid chains, but also of the ratio of the different lipids (Sohlenkamp and Geiger, 2016). For example, the proportion of unsaturated fatty acids varies with temperature to modify the fluidity of the membrane. Another example of adaptation is the increase of CL following increase of osmotic pressure. Also, in stationary phase, there is a relative increase in anionic phospholipids, together with the replacement of fatty acid unsaturations by cyclopropane groups. This conversion is performed by the Cfa enzyme, whose expression is activated by σ^S^ alternative Sigma factor (Wang and Cronan, 1994; Eichel et al., 1999). In addition, global transcriptome studies had suggested that expression of PL synthesis genes might be globally affected during the stringent response (Durfee et al., 2008), and that specific genes of PL synthesis might be regulated by envelope stress responses (De Wulf et al., 2002; Rhodius et al., 2006).

In bacteria, several regulating pathways are present to monitor and respond to envelope perturbations. In *E. coli*, the alternative Sigma factor σ^E^ responds to the accumulation of unfolded outer membrane proteins or altered forms of LPS in the periplasm through a proteolytic signaling cascade (Mitchell and Silhavy, 2019). In addition, two-component systems such as CpxRA, RcsBA, or BaeRS respond to various envelope perturbations such as defects in protein secretion, alterations in LPS or peptidoglycan, or exposure to toxic compounds, by a phosphorylation signaling cascade that ends up in the activation of a transcriptional regulator (Mitchell and Silhavy, 2019). We have previously shown that *plsB*, coding for the first acylation step in PL synthesis, is activated by the envelope stress response σ^E^ factor, and that *dgkA*, coding for a diacylglycerol kinase involved in PL recycling, is controlled by the two-component system BasRS (Wahl et al., 2011).

The *psd* gene coding for phosphatidylserine decarboxylase, the last step in phosphatidylethanolamine synthesis, appears in the regulon of σ^E^ like *plsB*, but also in the regulon of the CpxAR two-component system (De Wulf et al., 2002; Rezuchova et al., 2003; Rhodius et al., 2006). The *psd* gene is followed by *mscM* (previously called *yjeP*), and they probably form an operon. MscM protein is a miniconductance mechanosensitive channel of the MscS family, involved in the protection of the physical integrity of the cell during transitions from high to low osmolarity (Booth, 2014; Edwards et al., 2012).

In this study, we dissected the organization of the promoter region of the *psd*-*mscM* operon, and we studied the dual regulation of these genes by σ^E^ and the response regulator CpxR. We showed that the operon is under the control of two distinct promoters. One is activated by σ^E^, the other by CpxRA. This second promoter is responsible for basal expression of the operon. Therefore, the PL synthesis pathway (and more specifically the PE pathway) is controlled by envelope stress responses at both its first and last steps, which might be important for adaptation of the membrane to envelope perturbations.

## Materials and Methods

### Plasmid and Strain Constructions

pUA66 and pUA-psdP2 plasmids were obtained from the library of *E. coli* promoters fused to GFP coding sequence (Zaslaver et al., 2006). The other *psd* transcriptional fusions were constructed using primers indicated in Supplementary Table 1, and cloned in *Xho*I/*Bam*HI sites of pUA66. Expression plasmids for *rpoE*, *nlpE*, and *nlpE*_*IM*_ were constructed using primers indicated in Supplementary Table 1, and cloned in *Eco*RI/*Sal*I sites of pBAD24 vector (Guzman et al., 1995). A region of 1600 base pairs encompassing *psd* promoter was cloned in pKO3 vector (Link et al., 1997). Mutations were introduced in the pKO3-*psd* vector, in the pBAD-*nlpE* and in the transcriptional fusions by PCR mutagenesis on plasmid, using the oligonucleotides indicated (Table 1 and Supplementary Table 1).

**TABLE 1:** Plasmids.

**Lab code**	**Name**		**Description**	**References**
pEB0794	pJL148		ampR, kanaR, SPA-FRT-kanaR-FRT cassette	Zeghouf et al., 2004
pEB0267	pKD46		ampR, ts, lambdaRed genes	Datsenko and Wanner, 2000
pEB0266	pCP20		camR, ampR, pSC101 ori, ts	Cherepanov and Wackernagel, 1995
pEB0227	pBAD24		ampR, colE1 ori, PBAD promoter	Guzman et al., 1995
pEB1470	pBAD-*nlpE*		PCR ebm981/982 (*Eco*RI/*Xho*I) in pBAD24 (*Eco*RI/*Sal*I)	This work
pEB1966	pBAD-*nlpE*_*IM*_		PCR mutagenesis ebm1785/1786 on pEB1470	This work
pEB1102	pBAD-*rpoE*			Wahl et al., 2011
pEB0232	pKO3		camR, pSC101 ori, *sacB*	Link et al., 1997
pEB1973	pKO3-*psd*		PCR ebm1762/1763 (*Bgl*II/*Xho*I) in pEB0232 (*Bam*HI/*Sal*I)	This work
pEB1983	pKO3-*psd*_mutCpxR		PCR mutagenesis ebm1777/ebm1778 on pEB1973	This work
pEB1988	pKO3-*psd*_mutPσ^E^		PCR mutagenesis ebm1808/ebm1809 on pEB1973	This work
pEB2021	pKO3-*psd*_mutPσ^E^_mutCpxR		PCR mutagenesis ebm1777/ebm1778 on pEB1988	This work
pEB0067	pACYC184		camR, p15A ori	Chang and Cohen, 1978
pEB1975	pACYC-psd-3Flag		PCR ebm1762/968 on EB1075 (*Bgl*II/*Xho*I) in pACYC184 (*Bam*HI/*Sal*I)	This work
pEB2033	pACYC-psd(S254A)-3Flag		PCR mutagenesis ebm1911/1912 on pEB1975	This work
pEB2099	pACYC-psd		PCR ebm1762/1763 (*Bgl*II/*Xho*I) in pACYC184(*Bam*HI/*Sal*I)	This work
pEB0898	pUA66		kanaR, pSC101 ori, GFPmut2	Zaslaver et al., 2006
		Limits^*a*^		
pEB1120	psdBis	−391/+57	PCR ebm435/436 in pUA66 *Xho*I/*Bam*HI	This work
pEB1509	psdPσ^E^	−391/−229	PCR ebm435/1023 *Xho*I/*Bam*HI in pUA66	This work
pEB1981	psdPσ^E^mut		PCR mutagenesis ebm1808/1809 on pEB1509	This work
	psdP2	−196/+95		Zaslaver et al., 2006
pEB1963	psdP2mutCpxR		Mutagenesis ebm1777/ebm1778 on pUA-psdP2	This work

*^*a*^The limits of the transcriptional fusions are given relative to the start codon of *psd*.*

In order to introduce the sequence coding for the Flag or SPA tags downstream of the coding sequences of interest on the chromosome, we amplified the corresponding cassette from the template plasmid pJL148 (Zeghouf et al., 2004). MG1655_Psd-Flag and MG1655_MscM-SPA strains were then constructed by PCR recombination at the locus followed by P1 transduction as described previously (Datsenko and Wanner, 2000) (Table 2). When required, the gene conferring resistance to Kanamycin was removed using the pCP20 plasmid (Cherepanov and Wackernagel, 1995), allowing the transformation by transcriptional fusion plasmids. Point mutations were introduced on the chromosome using the pKO3 vector (Link et al., 1997).

**TABLE 2:** *Escherichia coli* strains.

**Lab code**	**Name**	**Description**	**References**
EB758	BW25113 Δ*cpxR*:kana^*R*^		Baba et al., 2006
EB944	MG1655	Wild-type reference. F- λ- rph-1	Lab stock
EB425	MG1655 ppGpp°	Δ*relA*Δ*spoT*::cat	Wahl et al., 2011
EB559	MG1655Δ*dksA*		Wahl et al., 2011
EB544	MG1655 ppGpp+	MG1655Δ*relA spoT203 zib563:tetra*	My et al., 2013
EB780	MG1655 Δ*cpxR*	P1 transduction from EB758 in MG1655. kanaR cassette removed using pCP20.	This work
EB533	EH150	Mutation Gly280Asp. *psd*-*2*^ts^	Hawrot and Kennedy, 1975
EB1075	psd-3Flag	PCR ebm472/448 on pJL148 recombined in MG1655pKD46, then P1 transduction in MG1655	This work
EB1078	Δ*cpxR psd*-3Flag	P1 transduction from EB1075 in EB780	This work
EB1098	Psd-3Flag_mutCpxR	Recombination pEB1983 in EB1075	This work
EB1085	Psd-3Flag_mutPσ^E^	Recombination pEB1973 in EB1075	This work
EB1113	Psd-3Flag_mutCpxRmutPσ^E^	Recombination pEB2021 in EB1075	This work
EB1095	MscM-SPA	PCR ebm488/489 on pJL148 recombined in MG1655pKD46, then P1 transduction in MG1655	This work
EB1081	Psd_mutCpxR	Recombination pEB1983 in MG1655	This work
EB1103	Psd_mutPσ^E^	Recombination pEB1988 in MG1655	This work
EB1112	Psd_mutCpxRmutPσα^E^	Recombination pEB2021 in MG1655	This work

## Transcriptional Fusions with GFP

We used several clones from the *E. coli* transcriptional fusions library (Zaslaver et al., 2006) and we constructed the required additional transcriptional fusions (see above for plasmid construction and Table 1). MG1655 wild-type *E. coli* strain or isogenic mutants were transformed with plasmids carrying the *gfp* transcriptional fusions and maintained with kanamycin. For co-transformation, compatible plasmids (pBAD24 and derivatives) were used, and ampicillin added. Selection plates were incubated at 37°C for 16 h. A total of 600 μl of LB medium supplemented with required antibiotics, and with 0.01% arabinose when necessary for pBAD-driven expression, were incubated (four to six replicate each assay) and grown for 16 h at 30°C in 96-well polypropylene plates of 2.2 ml wells under aeration and agitation. Fluorescent intensity measurement was performed in a TECAN infinite M200 plate reader. A total of 150 μl of each well was transferred into black Greiner 96-well plate for reading optical density at 600 nm and fluorescence (excitation: 485 nm; emission: 530 nm). The expression levels were calculated by dividing the intensity of fluorescence by the optical density at 600 nm, after subtracting the values of blank sample. These results are given in arbitrary units because the intensity of fluorescence is acquired with an automatic optimal gain and hence varies from one experiment to the other.

### SDS-PAGE and Western Blotting

Total cell extracts were prepared by resuspending cell pellets in Laemli buffer 1X at a concentration of 0.3 uOD_600__nm_ in 10 μl, and then heating for 10 min at 95°C. After separation of 8 μl of total cell extracts on SDS-PAGE, electrotransfer onto nitrocellulose membranes was performed using Trans-Blot turbo transfer system from Bio-Rad. After blocking in PBS 1X + milk 5%, SPA-tagged or 3Flag-tagged proteins were detected with monoclonal anti-Flag M2 antibody purchased from Sigma. IscS protein was used as an internal control and revealed with polyclonal anti-IscS antibodies (Gully et al., 2003). Fluorescent secondary antibodies were respectively IRDye 800 anti-mouse and IRDye 680 anti-rabbit purchased from Li-Cor. Scanning and quantification were performed on a Li-Cor Odyssey-Fc imaging system, reading at 700 nm (for IscS detection) or 800 nm (for Flag detection).

### Reverse Transcriptase – PCR

RNAs were purified using the NEB Monarch Total RNA MiniPrep Kit and further digested with Dnase I followed by clean up with the Qiagen Rneasy Mini kit. Reverse transcription was performed using Invitrogen SuperScript III First-Stand Synthesis System with random hexamers. Finally, PCR was performed using ebm446 and ebm2079 primers.

## Results

**FIGURE 1:**
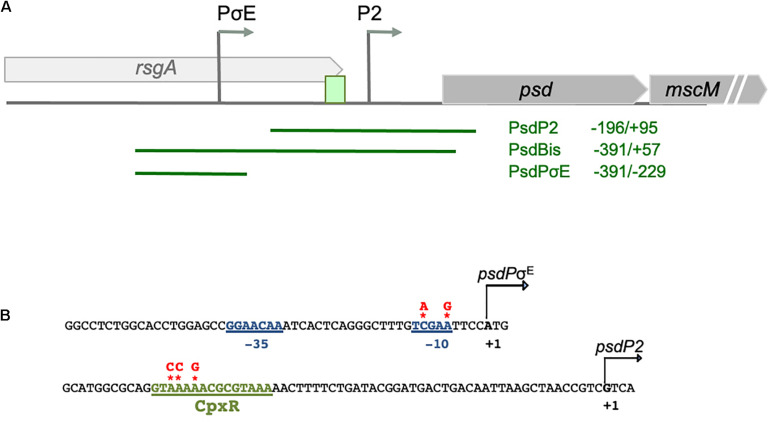
Promoter region of *psd*-*mscM* operon. **(A)** Positions of the predicted CpxR box, and of the two transcription start sites are indicated by a green box and gray arrows. Limits of the regions cloned in transcriptional fusion with gfp, in the pUA66 plasmid, are shown by green horizontal lines. End positions are given relative to the start codon of *psd* gene. **(B)** DNA sequences of the two promoters. Mutations introduced in the promoter regions or in the CpxRbox are indicated at the top of red stars.

In a global experimental study of all the transcriptional starting sites of *E. coli* (Thomason et al., 2015), two putative promoters were identified upstream the *psd* ORF (Figure 1). The first one, that we will call psdPσ^E^, had been already reported as a promoter activated by the alternative sigma factor σ^E^ (Rezuchova et al., 2003; Rhodius et al., 2006). The second one, that we will call psdP2, is located 197 nucleotides downstream psdPσ^E^. It has already been reported that *psd* expression was activated by CpxR, and a putative CpxR box was proposed (De Wulf et al., 2002). This CpxR box is located 41 nucleotides upstream the predicted transcription start site of the psdP2 promoter, which is a distance in perfect agreement with CpxR acting as an activator of the psdP2 promoter (Busby, 2019).

### Dissection of the Promoter Region

In order to dissect the promoter region of *psd* and to study the genetic regulation of *psd* expression, we first used transcriptional fusions with GFP, expressed from the low copy vector pUA66, which enable to follow promoter activity by measuring fluorescence directly in living cells (Zaslaver et al., 2006). A transcriptional fusion containing the upstream region of *psd* ORF was already available from a library of *E. coli* promoters (Zaslaver et al., 2006), but it only contained the putative psdP2 promoter. Therefore, we constructed additional transcriptional fusions, containing both promoters (called psdBis) or only the psdPσ^E^ promoter (Figure 1A).

**FIGURE 2:**
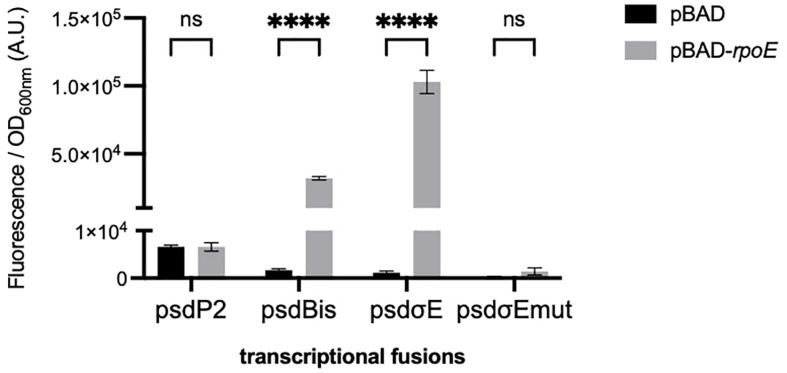
Effect of σ^E^ overproduction on the psdPσ^E^ promoter. MG1655 strain was transformed with plasmids carrying the transcriptional fusions pUA66, pUA-psdP2, pUA-psdBis, pUA-psdPσ^E^, or pUA-psdPσ^E^mut together with plasmids pBAD24 or pBAD-*rpoE*. Cultures were grown overnight at 30°C in LB supplemented with ampicillin, kanamycin, and 0.01% arabinose. The values show the mean ratio of GFP fluorescence over optical density at 600 nm, after subtraction of the pUA66 negative control, in arbitrary units (A.U.). The values are the mean of six replicas and the error bars show the SEM (standard error of the mean). ns, non-significant, ****p < 0.0001 in a two-way ANOVA statistical analysis.

We first studied the control of *psd* expression by σ^E^. We artificially induced the σ^E^ response, by overproducing the σ^E^ factor from an inducible pBAD-*rpoE* plasmid as previously described (Wahl et al., 2011). We co-transformed an *E. coli* wild-type strain with the different GFP transcriptional fusions and with the pBAD-*rpoE* plasmid. Upon σ^E^ overproduction, only the transcriptional fusions containing the psdPσ^E^ promoter (psdBis and psdPσ^E^, but not psdP2) showed a strong induction (Figure 2). We then mutated two nucleotide positions in the predicted −10 box of the psdPσ^E^ promoter. These mutations completely abolished the induction of the psdPσ^E^ transcriptional fusion by pBAD-*rpoE* (Figure 2).

**FIGURE 3:**
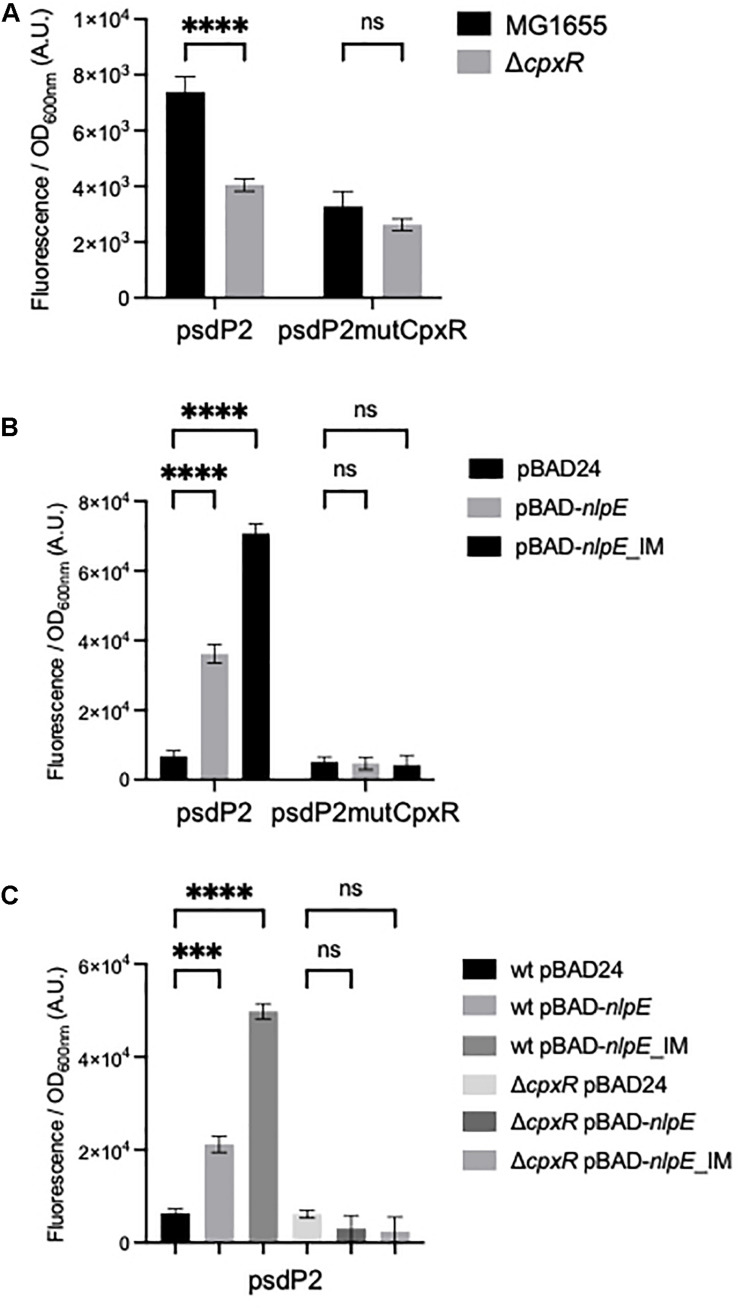
Control of the psdP2 promoter by CpxR. **(A)** MG1655 or Δ*cpxR* strains were transformed with the indicated transcriptional fusions. Cultures were grown overnight at 30°C in LB supplemented with kanamycin. **(B,C)** MG1655 or Δ*cpxR* strains were transformed with the indicated transcriptional fusions together with pBAD24, pBAD-*nlpE*, or pBAD-*nlpE_IM* plasmids. Cultures were grown overnight at 30°C in LB supplemented with ampicillin, kanamycin and 0.01% arabinose. The values show the mean ratio of GFP fluorescence over optical density at 600 nm, after subtraction of the pUA66 negative control, in arbitrary units (A.U.). The values are the mean of 12 **(A)** or 4 **(B,C)** replicas and the error bars show the SEM (standard error of the mean). ns, non-significant, ***p < 0.001, ****p < 0.0001 in a two-way ANOVA statistical analysis.

We then tested if CpxR was a transcriptional activator of the psdP2 promoter. We first compared the activity of the psdP2 transcriptional fusion in wild-type and Δ*cpxR* strains. This activity was reduced in the Δ*cpxR* strain (Figure 3A), suggesting a potential role of CpxR in the regulation of *psd* expression, even in balanced growth conditions. In reverse, in order to induce the CpxR response, we overproduced the NlpE lipoprotein or its NlpE_IM_ variant that triggers an even stronger response (Delhaye et al., 2016). We co-transformed an *E. coli* wild-type strain by the different transcriptional fusions and by the pBAD-*nlpE* or -*nlpE*_*IM*_ plasmids. The psdP2 transcriptional fusion showed an activation when NlpE was overproduced, and the effect was reinforced with NlpE_IM_ (Figure 3B). Furthermore, the activation was abolished when the experiment was repeated in a Δ*cpxR* strain (Figure 3C), which showed that this was indeed the result of an activation of the CpxR pathway. Finally, to prove that the activation was due to direct binding of CpxR response regulator to the psdP2 promoter, we introduced point mutations that destroy the CpxR binding motif in the psdP2 transcriptional fusion (Figure 1B). With this mutated transcriptional fusion, the activation of psdP2 promoter was abolished (Figure 3B).

In conclusion, *psd* expression (and potentially *mscM* expression) is under the control of two distinct promoters. One is activated by σ^E^, the other by CpxRA. Given that this promoter organization is very similar to the one we described previously for *plsB* (Wahl et al., 2011), we hypothesized that this second promoter might be regulated by ppGpp as shown for *plsB* and suggested by global study of the stringent response (Durfee et al., 2008). We tested this by measuring the activity of the transcriptional fusions in strains with modified ppGpp levels as previously described (Wahl et al., 2011). The ppGpp° (Δ*relA*Δ*spoT*) strain is devoid of ppGpp, the DksA cofactor is necessary for ppGpp action on RNAP hence a Δ*dksA* mutant mimics the absence of ppGpp, and finally the *spoT203* mutant is impaired in ppGpp degradation, leading to ppGpp accumulation in the cell. The psdP2 promoter did not show a strong dependence on ppGpp levels (Supplementary Figure 1). The psdP2 expression did slightly increase in a Δ*dksA* mutant, but not in the ppGpp° mutant, and it did not show a corresponding repression in the ppGpp+ mutant. Interestingly, the activity of the psdPσ^E^ transcriptional fusion increased in the ppGpp accumulating mutant (Supplementary Figure 1), which is in agreement with the known positive effect of ppGpp on σ^E^ (Costanzo et al., 2008).

### Impact of σ^E^ or CpxR Regulation on Psd and MscM Protein Amounts

**FIGURE 4:**
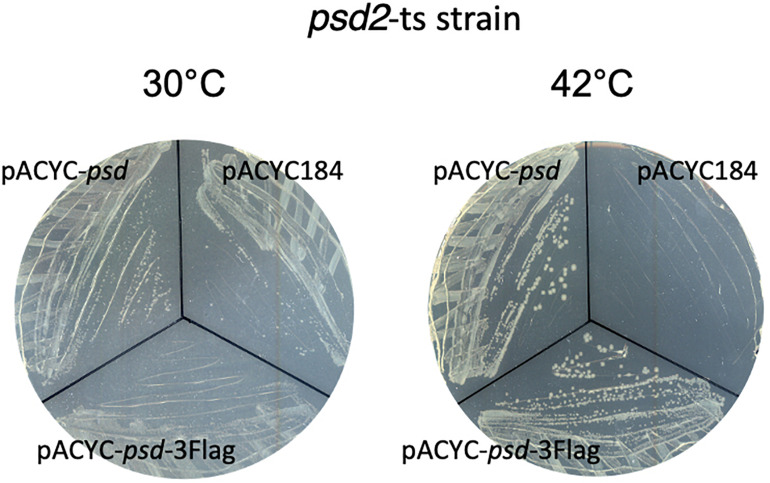
Strain EH150 *psd2*-ts (Hawrot and Kennedy, 1975) was transformed by pACYC184, pACYC-*psd*, or pACYC-psd-3Flag plasmids and the selection was performed at 30°C. Then, colonies were spread out on LB chloramphenicol plates at 30 or 42°C for 24 h.

**FIGURE 5:**
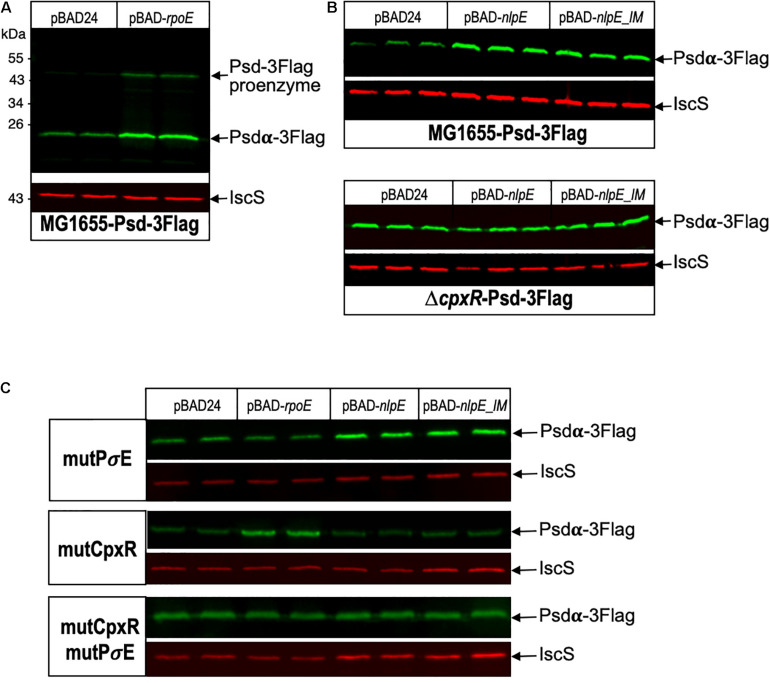
Psd protein levels are controlled by σ^E^ and CpxR. **(A)** *E. coli* Psd-3Flag strain was transformed by pBAD24 or pBAD-*rpoE* plasmids. Two replicas of each transformation were grown in LB supplemented with ampicillin until OD_600__nm_ = 0.8, then cultures were induced with 0.2% arabinose for 2 h. **(B)** Psd-3Flag and Δ*cpxR*-Psd-3Flag strains were transformed by pBAD24, pBAD-*nlpE*, or pBAD-*nlpE_IM* plasmids. Three replicas of each transformation were grown in LB supplemented with ampicillin until OD_600__nm_ = 0.8, then cultures were induced with 0.2% arabinose for 2 h. **(C)** Psd-3Flag_mutPσ^E^, Psd-3Flag_mutCpxR, and Psd-3Flag_mutCpxRmutPσ^E^ strains containing mutations in the promoter region were transformed by pBAD24, pBAD-*rpoE*, pBAD-*nlpE*, or pBAD-*nlpE_IM* plasmids. Two replicas of each transformation were grown in LB supplemented with ampicillin until OD_600__nm_ = 0.8, then cultures were induced with 0.2% arabinose for 2 h. Proteins were separated by SDS-PAGE 12% and detected by western blot using anti-Flag monoclonal antibody to detect Psd-3Flag or anti-IscS polyclonal antibody as an internal loading control. Quantification of the bands are shown in Supplementary Figure 4.

Because *psd* expression is controlled by two promoters, we then wanted to study the relative involvement of these two promoters in the control of the amounts of Psd in the cell. For that, we constructed a strain where Psd is fused at its C-terminus with the 3Flag tag, in order to detect the protein by western blot with a monoclonal antibody. Psd is produced as a proenzyme, which after endoproteolysis gives rise to a mature two-domains enzyme (Li and Dowhan, 1990; Choi et al., 2015). Because the tag is fused to the C-terminus of the protein, we detected as expected the small C-terminal Psdα-3Flag subdomain, indicating that the cleavage occurred normally (it migrated at a higher position than the expected 12 kDa, but this is not exceptional, and might be influenced by the pyruvoyl N-terminal modification). Furthermore, the tagged strain did not display any obvious growth defect, suggesting that the Psd-3Flag fusion was functional. However, in order to prove the functionality of the Psd-3Flag tagged protein, we also cloned this construction or the wild-type *psd* gene under its own promoter in a plasmid, and tested the complementation of a *psd*-ts mutant (Hawrot and Kennedy, 1975). Expression of *psd*-3Flag or wild-type *psd* expressed from a plasmid similarly restored growth of the *psd*^ts^ mutant at 42°C, showing that the recombinant Psd-3Flag protein was functional (Figure 4). We then tested the effect of overproducing σ^E^ or inducing the CpxR response through NlpE expression. The levels of Psd-3Flag protein were clearly increased when σ^E^ was overproduced (Figure 5A). Similarly, levels of Psd-3Flag increased when NlpE or NlpE_IM_ proteins were overproduced (Figure 5B). The increases observed when the CpxRA pathway was artificially induced were not as strong as the ones observed when σ^E^ was overproduced. However, they were clearly abolished when the experiment was repeated in a Δ*cpxR* genetic context (Figure 5B). It has to be noted that when Psd-3Flag amounts were strongly increased, a band at approximately 45 kDa was also detected (Figure 5A). We hypothesized that this band might correspond to the accumulation of an unmatured full length Psd-3Flag proenzyme. In order to test this, we introduced the point mutation S254A, which is known to prevent the maturation of Psd (Li and Dowhan, 1990), in the plasmid expressing *psd*-3Flag. As expected, this mutant form was detected at 45 kDa (Supplementary Figure 2). Interestingly, when we compared the production of Psd-3Flag with or without induction of *rpoE*, the band at 45 kDa accumulating when *rpoE* is induced migrated at the exact same size than the unmatured Psd(254A)-3Flag (Supplementary Figure 2), suggesting that endoproteolytic cleavage is indeed impaired when Psd-3Flag is overproduced.

**FIGURE 6:**
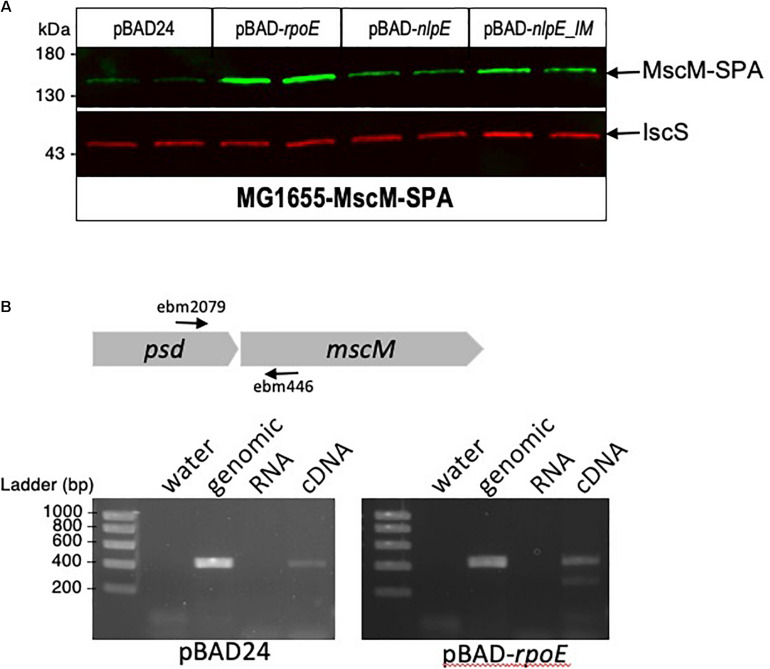
*mscM* is in operon with *psd*, under control of the σ^E^ and CpxR dependent promoters. **(A)** *E. coli* MscM-SPA strain was transformed by pBAD24, pBAD-*rpoE*, pBAD-nlpE, or pBAD-*nlpE_IM* plasmids. Two replicas of each transformation were grown in LB supplemented with ampicillin until OD_600__nm_ = 0.8, then cultures were induced with 0.2% arabinose for 2 h. Proteins were separated by SDS-PAGE 10% and detected by western blot using anti-Flag monoclonal antibody for the detection of MscM-SPA or anti-IscS polyclonal antibodies as an internal loading control. **(B)** RT-PCR was performed on MG1655 strain transformed by pBAD or *pBAD-rpoE* induced in the above conditions. The black arrows indicate the positions of the primers used for the amplification. PCR controls on water, genomic DNA, and equivalent amounts of RNA as in the RT-PCR reaction were performed.

On the genome of *E. coli*, *psd* ORF is followed by *mscM* ORF, suggesting an organization of these two genes as an operon. To study the involvement of σ^E^ and CpxR on *mscM* expression, we constructed a strain where MscM is fused at its C-terminus with the SPA tag. MscM-SPA was faintly detected on Western blot at the expected size of 130 kDa, and MscM protein levels were increased when *rpoE* or *nlpE* were artificially expressed, similarly as for Psd-3Flag (Figure 6A). We then performed an RT-PCR experiment, which confirmed that *psd* and *mscM* are expressed as an operon, controlled by the same two promoters (Figure 6B).

### Characterization of Strains Devoid of *psd*-*mscM* Control by σ^E^ and CpxR

The above experiments using transcriptional fusions or following the amount of Psd and MscM proteins demonstrated the control of *psd* and *mscM* expression by the two envelope stress response pathways mediated by σ^E^ or CpxR. The next question was naturally to understand the physiological role of these regulations. To address this question, we chose to introduce point mutations in the promoters of *psd*, on the chromosome, in order to prevent specifically the activation of *psd*-*mscM* expression by σ^E^, CpxR or both stress responses at the same time. We have shown above that the mutations chosen in the σ^E^ promoter and CpxR box completely abolished the induction of the transcriptional fusions by pBAD-*rpoE* or pBAD-*nlpE*, respectively (Figures 2, 3). Note that both mutations are localized inside the ORF of the upstream *rsgA* gene. The mutation in the PsdPσ^E^ promoter does not modify the amino acid sequence of RsgA, while the mutation in the CpxR box does modify residue Lys340 of RsgA in Glutamine. We introduced the mutations in wild-type MG1655 strain and in the Psd-3Flag strain using homologous recombination with the pKO3 plasmid (Link et al., 1997). The three types of mutants were readily obtained in the two strains (Table 2). The mutant strains did not display any obvious growth phenotype in LB (Supplementary Figure 3) showing that σ^E^ and CpxR regulations are not essential in balanced growth conditions. In the Psd-3Flag background strains, we observed that mutations in the Pσ^E^ promoter and CpxR box had no effect on the basal amounts of Psd-3Flag protein (Figure 5C and data not shown). This suggests that the decreased expression observed with the psdP2muCpxR transcriptional fusion (Figure 3A) does not impact protein level, and shows that these regulations are not important to control basal Psd and MscM protein levels. However, when σ^E^ was induced, the amounts of Psd-3Flag protein did not increase anymore in the strains containing the Pσ^E^ mutation (Figure 5C, first and third panel), while Psd-3Flag still increased in the CpxRbox mutant (Figure 5C, second panel). When the CpxRA pathway was induced, with pBAD-*nlpE* or pBAD-*nlpE_IM* plasmids, Psd-3Flag protein amounts did not increase anymore in the strains containing the mutation in the CpxR binding site (Figure 5C, second and third panel), while Psd-3Flag still increased in the Pσ^E^ mutant (Figure 5C, first panel). These results confirmed what we observed with the transcriptional fusions (Figure 3A), and showed that the σ^E^ and CpxR control of *psd* promoters impact the amount of proteins produced *in vivo* upon stress induction.

## Discussion

The goal of this work was to decipher the genetic control of *psd*-*mscM* operon and its regulation by the two envelope stress responses mediated by the alternative Sigma factor σ^E^ and by the two-component system CpxRA. We found that *psd* promoter region comprises two promoters, confirming the +1 transcription start sites identified previously (Thomason et al., 2015): a distal σ^E^ promoter and a proximal P2 promoter, activated by CpxRA, which is likely to be also responsible for the basal expression of *psd*. This organization is very similar to what we observed previously for the promoter of *plsB*, which comprises a distal σ^E^ promoter and a proximal P2 promoter, responsible for the basal expression of *plsB* (Wahl et al., 2011). However, *plsB* expression was not controlled by CpxR (data not shown). We also systematically tested if other genes of PL synthesis were regulated by σ^E^ or CpxR but it was not the case (data not shown). Therefore, it appears that envelope stress responses control the first step of PL synthesis (*plsB*) and the last step of PE synthesis (*psd*). This suggests that in response to envelope stress, an increase in the pathway for PE might be important, in order to help in envelope biogenesis processes such as LPS or outer membrane proteins trafficking. An enhanced PE biosynthesis might be obtained by “pushing” from the top with PlsB and “pulling” from the bottom of the pathway with Psd. However, enzymes of PL synthesis are believed to be present in excess and it is not clear how increasing enzyme amounts might have an effect on the flux in the PL synthetic pathway. This regulation might be a long-term adaptation rather than an immediate response to the encountered stress. An alternative hypothesis is that the enzymes might be damaged during envelope stress and need to be replaced.

We previously showed that the basal promoter of the *plsB* gene was repressed by ppGpp (Wahl et al., 2011). Furthermore, it was proposed that the genes for PE pathway, including *psd*, might be downregulated during the stringent response, while the genes for PG/CL were upregulated (Durfee et al., 2008), which would be consistent with an increase in PG and CL in the membrane during growth arrest. However, we did not detect a strong regulation of psdP2 promoter by ppGpp (Supplementary Figure 1).

It is striking to find *psd* in operon with a gene encoding a mechanosensitive channel. There is no obvious direct functional link between these two genes, apart from a function in membrane homeostasis. Mechanosensitive channels open in response to transition from high to low osmolarity in order to protect cells from bursting (Booth, 2014). There are seven mechanosensitive channels in *E. coli*, MscL and six channels of the MscS family, including MscM. This redundancy might be explained by a need to respond to different rates or intensities in osmolarity variations (Booth, 2014). As for *psd*, a genetic regulation might appear irrelevant for a rapid response to physical stress. However, the number and types of channels are important factors to determine the protective ability and cell fate (Bialecka-Fornal et al., 2015), and it has been shown that over-expression of MscM confers protection to hypo-osmotic shock in a strain otherwise devoid of all the other mechanosensitive channels (Edwards et al., 2012). Therefore, increased expression of *mscM* by σ^E^ and CpxR in response to envelope stress might be a bet hedging strategy, as it might confer adaptation for future additional envelope injuries. Similarly, *mscL* and *mscS* genes are regulated by the alternative Sigma factor σ^S^ (Stokes et al., 2003). Also, in *Pseudomonas aeruginosa*, the *cmpX* gene coding for a putative mechanosensitive channel is part of the SigX envelope stress response regulon (Gicquel et al., 2013). Furthermore, these mechanosensitive channels possess diverse periplasmic and cytoplasmic domains that might play additional functions in stress responses, which for example was suggested recently for YbdG in *E. coli* (Amemiya et al., 2019).

Unfortunately, we were not able yet to find growth conditions or stress conditions where the σ^E^ or CpxRA regulations on *plsB* and *psd-mscM* had a role for cell physiology. We tried to subject the mutant strains to stresses known to induce the σ^E^ or CpxRA pathways, i.e., osmotic stress, alkaline stress, envelope porin misfolding or mislocalization, or heat shock, but we did not detect any effects in these experiments. As mentioned above, these regulations are certainly involved in long-term adaptation rather than being an immediate response to the encountered stress. This might be why we did not detect any obvious phenotypic defects in the regulatory mutant strains when testing for immediate resistance to stress. Combining stresses, long-term, or competition experiments might be needed to be able to unravel a phenotypic advantage of the wild-type over the regulatory mutant strains. Interestingly, and in a reversed way, several studies have shown that PL synthesis mutants, which affect membrane composition, activate envelope stress responses, including σ^E^ and CpxRA signaling pathways (Mileykovskaya and Dowhan, 1997; Itou et al., 2012; Rowlett et al., 2017; Nepper et al., 2019). Therefore, the σ^E^ or CpxRA regulations of *plsB* and *psd-mscM* might also be part of a feedback loop involved in maintaining an optimum PL synthesis balance for membrane homeostasis.

## Data Availability Statement

The original contributions presented in the study are included in the article/Supplementary Material, further inquiries can be directed to the corresponding author/s.

## Author Contributions

YH, EB, and JV contributed to conception and design of the study. YH, JB, and AW performed experiments. EB wrote the manuscript. All authors contributed to manuscript revision, read, and approved the submitted version.

## Conflict of Interest

The authors declare that the research was conducted in the absence of any commercial or financial relationships that could be construed as a potential conflict of interest.
