# Supplementary material for: Dual Regulation of Phosphatidylserine Decarboxylase Expression by Envelope Stress Responses
Source: Front Mol Biosci. 2021 May 7;8:665977. doi: 10.3389/fmolb.2021.665977 (PMC8138132; doi:10.3389/fmolb.2021.665977)
Supplement: Supplementary file 1 [file Data_Sheet_1.PDF]

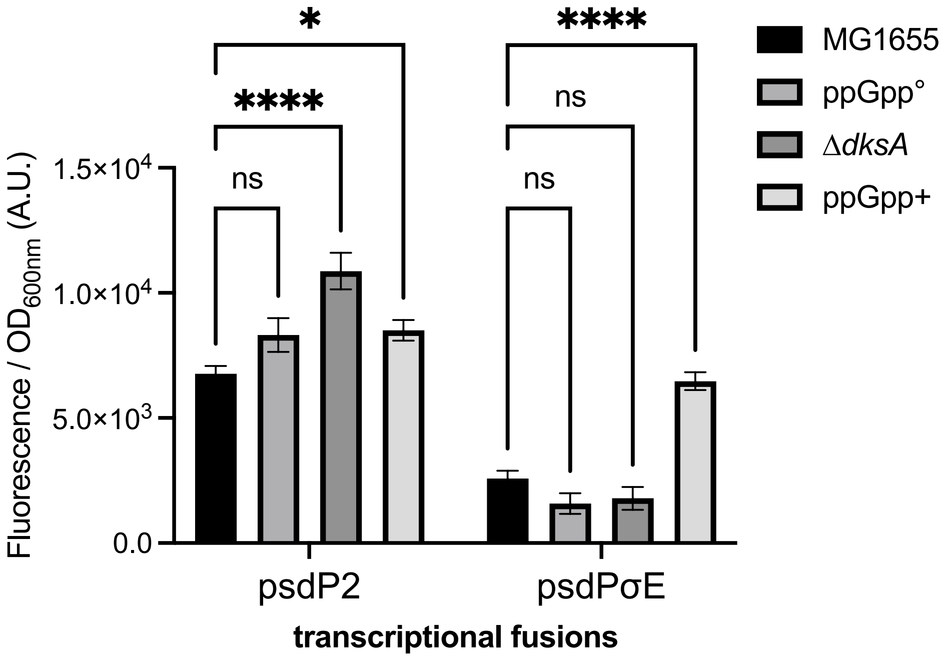

Figure S1 : Effect of ppGpp on the activity of the *psd-mscM* promoters.
MG1655, ppGpp° (Δ*relA*Δ*spoT*), Δ*dksA*, and ppGpp+ (Δ*relAspoT203*) strains were transformed with the indicated transcriptional fusions. The culture were grown overnight at 30°C in LB supplemented with kanamycin. The values show the mean ratio of GFP fluorescence over optical density at 600 nm, in arbitrary units (A.U.). The values are the mean of 6 replicas. The error bars show the standard error of the mean. ns : non-significant, * : $p < 0.05$, ****: $p < 0.0001$ in a two-way ANOVA statistical analysis.

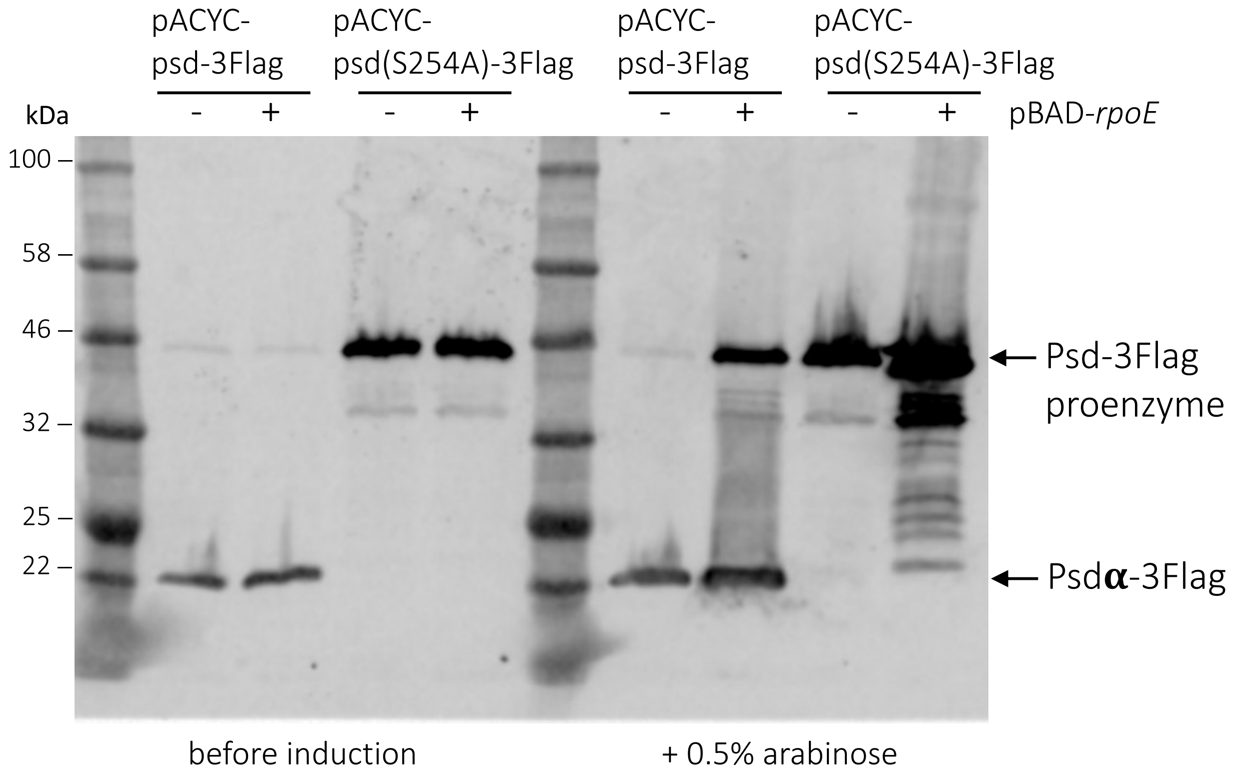

Figure S2. Accumulation of Psd-3Flag proenzyme. MG1655 *E. coli* wild type strain was co-transformed by pBAD24 or pBAD-*rpoE* plasmids and pACYC-psd3Flag or pACYC-psd(S254A)3Flag plasmids. Cultures were grown in LB supplemented with ampicillin until OD_{600nm}=0.7 then plasmid expression was induced with 0.5% arabinose for 2 hours. Proteins were separated by 12% SDS-PAGE and detected by Western-Blot using anti-Flag monoclonal antibody.

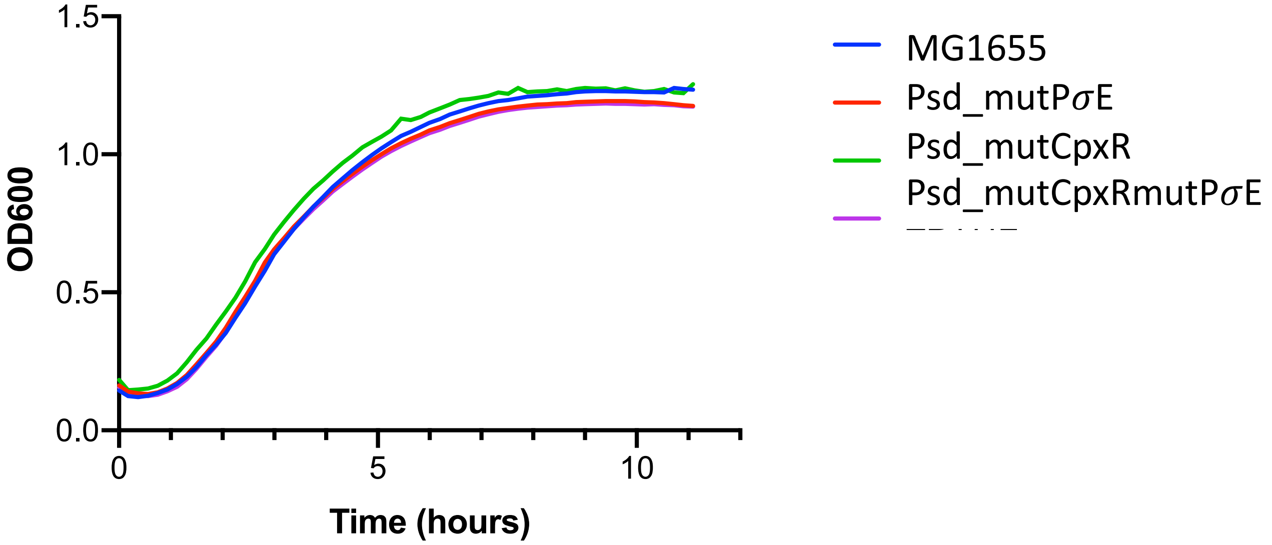

Figure S3. Growth of the regulation mutant strains. MG1655, Psd_mutPσE, Psd_mutCpxR, and Psd_mutCpxRmutPσE strains were grown in 96-well plates in 150 μl LB at 37°C in a TECAN M200 microplate reader. Lines represent the mean of 6 replicas for each strain.

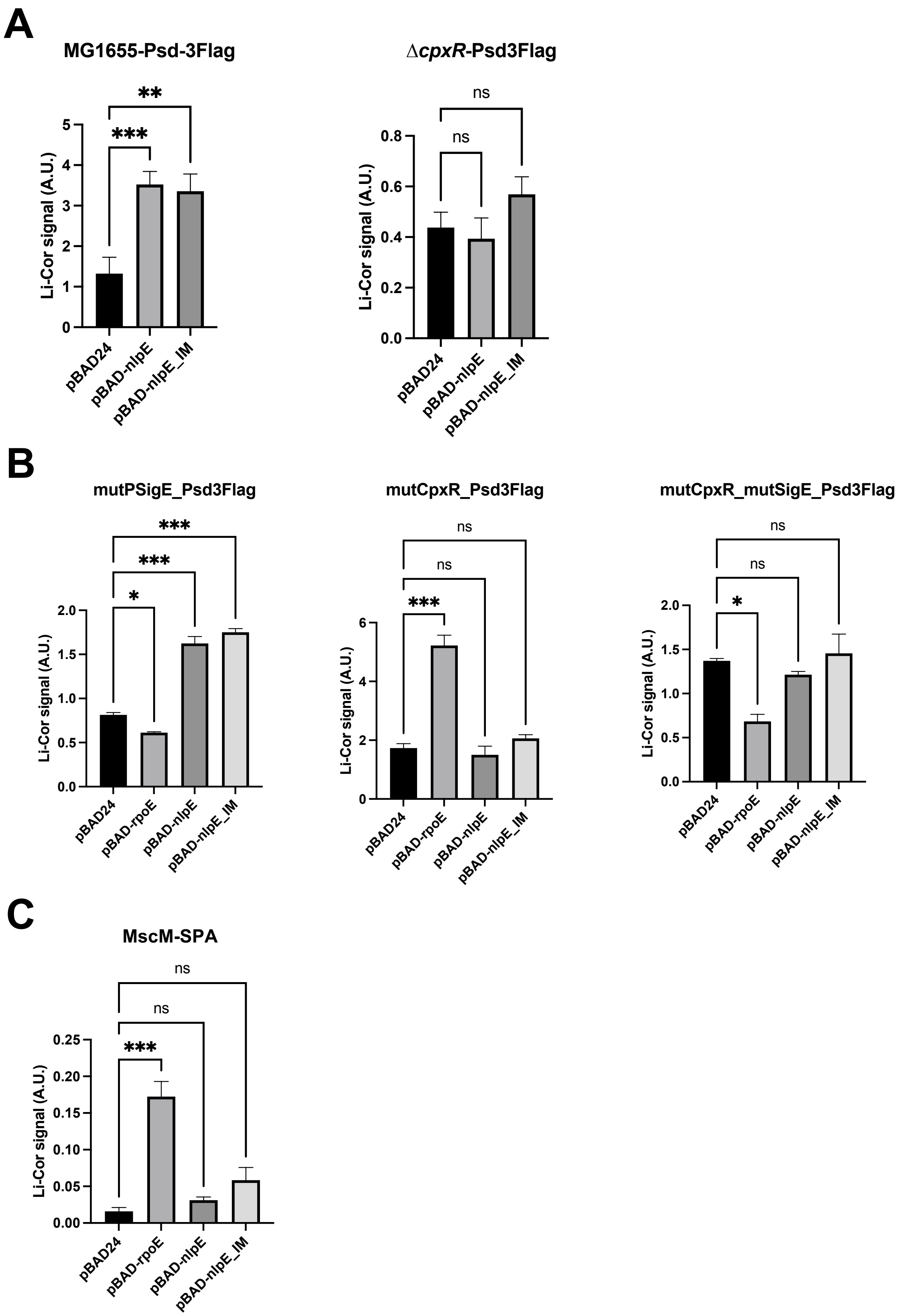

Figure S4. Quantification of the Psdα-3Flag bands of the Western blots of Figures 5 and 6. One-way ordinary ANOVA statistical tests were performed. However, note that these quantifications were performed on only 2 replicas for panels B and C, which relativizes the scope of the statistic analyses on this kind of data.

Table S1 : Oligonucleotides

Lab code	5’ - 3’ sequence	Name
Ebm435	ccg**ctcgag**TCGGCTGTATCACTTCCCGC	Prom psd FW
Ebm436	cg**agatct**GCGAGTAAGCCATAGTTTCGGC	Prom psd RV
Ebm446	TCTACGACTTCCGGCTGTGC	DOWN psd RV
Ebm448	CAGAAAAGTGATAATCAGGCGCACGTCAGCGTTTCCTTTGATGGAC ATATGAATATCCTCCTTAG	Psd-3Flag RV
Ebm472	GAACACGACGCCAGCCCATTGGTTGACGACAAAAAAGACCAGGTCA TTCCAACTACTGCTAGC	psd-3Flag FW
Ebm488	AGAACGCTGACGTCTGCGGGCAAAGGTCGTCAGGCGGGAAGTTTGT CCATGGAAAAGAGAAG	MscM-SPA FW
Ebm489	ATCAGTTTTGTTTGTGAGCCGGATTGGTTCATCCGGCACACAAACC ATATGAATATCCTCCTTAG	MscM-SPA RV
Ebm968	ttg**ctcgag**AAGCAGCTCCAGCCTACACG	RV P1pKD13
Ebm981	acc**gaattc**atgGTGAAAAAAGCGATAGTGAC	nlpE ORF FW
Ebm982	ttg**ctcgag**ttaCTGCCCCAAACTACTGCAATC	nlpE ORF RV
Ebm1023	cg**ggatcc**TCGGTATCGTGTTTGCAATCGC	Psd PsigE RV
Ebm1762	cg**agatct**AAGCGGTGCATGAGCGTACC	UP psd FW
Ebm1763	ccg**ctcgag**ttaGACCTGGTCTTTTTTGTCGTCAAC	psd ORF RV
Ebm1777	GGAAAGCATGGCGCAGGTccAgACGCGTAAAAACTTTTCTG	psd mutCpxR FW
Ebm1778	CAGAAAAGTTTTTACGCGTcTggACCTGCGCCATGCTTTCC	psd mutCpxR RV
Ebm1785	TTACTCTGATGGGATGT**G**ATAATCGGGCCGAAGTCGATAC	nlpE N22D FW
Ebm1786	GTATCGACTTCGGCCCGATTAT**C**ACATCCCATCAGAGTAA	nlpE N22D RV
Ebm1808	caaatcactcagggctttgtAgaGttccaTGACTATTTAGGTCTG	psd mutPσ^E FW
Ebm1809	CAGACCTAAATAGTCAtggaaCtcTacaaagccctgagtgatttg	psd mutPσ^E RV
Ebm1911	GTCGCTTTAAACTCGGcgCCACCGTTATCAACCTG	psd S254A FW
Ebm1912	CAGGTTGATAACGGTGGcgCCGAGTTTAAAGCGAC	psd S254A RV
Ebm2079	GAAAACGACGGTTCTGTGGC	UP mscM FW
